# Possible Association of Smokeless Tobacco Dependent Impairment in the Erythrocytes and Platelets Membranes of Human Male Volunteers: An Observation

**DOI:** 10.31557/APJCP.2019.20.7.2167

**Published:** 2019

**Authors:** Fareeda Begum Shaik, G Nagajothi, K Swarnalatha, C Vinod kumar, K Narender Dhania, C Suresh Kumar, Narendra Maddu

**Affiliations:** 1 *Department of Biochemistry, Sri Krishnadevaraya University, Ananthapuramu-, Andhra Pradesh,*; 2 *Department of Corporate Secretary Ship, Queen Mary’s College (Autonomous), Chennai, Tamil Nadu,*; 3 *Laboratory of Insect Molecular Biology and Biotechnology, Department of Animal Biology, School of Life Sciences, University of Hyderabad, Hyderabad, Telangana, India. *

**Keywords:** Smokeless tobacco- erythrocyte and platelet membrane- fluidity- iNOS- caspases

## Abstract

**Background:**

Smokeless tobacco (SLT) acts as a modifier of erythrocyte and platelet membranes by disrupting antioxidant system with the concomitant increase in free radical production and induction of apoptosis.

**Methods:**

The SLT users was that individuals used gutkha and khaini products (Khaleja/mahak chaini brand respectively) habitually, at least >20 times per week consists of 50-60 g during the last 2-4 years.

**Results:**

The gutkha and khaini users found to be significantly increased levels of iNOS (Inducible nitric oxide synthase) enzyme in plasma, erythrocytes, and platelet membranes when compared to normal controls. The gutkha and khaini users exhibited that the significant increase in the levels of gene expression of apoptotic proteins (Bcl_2_-B cell lymphoma gene 2, Bax, caspases 8, caspase 10, and caspase 12), IL-6 (Interleukin-6), and decreased levels of TNF-α (Tumor necrosis factor-alpha) and decreased expression of caspase 12 of khaini users were observed from blood samples. The significant increase in the concentrations of peroxynitrites (ONOO-), nitric oxide (NO) (Nitrates and nitrites), malondialdehyde (MDA), cholesterol, and phospholipids were reported in the smokeless tobacco users of erythrocytes and platelets. The experimental subjects showed that the increased osmotic fragility and decreased membrane fluidity of erythrocytes and platelets in comparison with non-tobacco users. The normal subjects had been exposed that the proper functioning of antioxidant enzymes and decreased enzyme activities of antioxidants were reported by SLT users.

**Conclusion:**

The smokeless tobacco products are exerted chronic damage to membranes of erythrocytes and platelets and elevation of apoptosis in the prolonged periods of human male volunteers.

## Introduction

Smokeless tobacco is one type of tobacco product, globally consumed and its prevalence is higher among youth. Nicotine is the major alkaloid present in the tobacco products in the form of (S) (−) nicotine and conversion of enantiomeric forms could not occur during the processing of tobacco leaves (Crooks et al., 1992; Zhang et al., 2018). During the recent years, in India, tobacco chewing is in the form of gutkha is alarmingly increasing among young adults (Avasn Maruthit et al., 2004). The components are widely used in the manufacture of smokeless tobacco exhibited equal or same nicotine availability and dependency (Croucher et al., 2013). According to International Agency for Research on Cancer (IARC), tobacco-specific N-nitrosamines (TSNA) like 4-methyl nitrosamino 1, 3 pyridyl butanone (NNK), nitrosonornicotine (NNN), nitrosoanatabine (NAT), and nitrosoanabasine (NAB) act as major nitrosamines are known carcinogens present in the smokeless tobacco products (Stanfill et al., 2015). The detectable concentrations of tobacco-specific nitrosamines are measured in the smokeless tobacco users indicated that the specificity maker of tobacco exposure and consumption (Brunnemann et al., 1996).

Gutkha is a SLT product and is markedly available in khaleja brand and khaini is mahak chaini/khaini SLT brand is available in small filter pouches of sachet across the India. The composition of gutkha is tobacco, areca nut, catechu, lime, saffron, and added flavours and khaini is the combination of tobacco and lime stone paste. Tobacco control policies could be considering the positive correlation between SLT consumption and widespread socio-economic inequities across the states in India (Thakur et al., 2015). The consumption of smokeless tobacco occurs as a worldwide habitual chewing, and consists of 237.4 million people were recorded in the India (Sinha et al., 2015). The decrease in consumption of smoking cigarettes during 2002-2012 was compensated by increased usage of SLT products aged ≥18 years adults (Agaku and Alpert, 2016).

In the countries of South-East Asia, people believed that the products of smokeless tobacco are less harmful than smoking cigarettes due to misleading advertising by tobacco companies (Suliankatchi et al., 2017). The levels of carcinogens and toxicants differ from one product to another and tobacco industries are designed to promote the manufacture of low carcinogen content in the SLT products (Borgerding et al., 2012). The enzyme of inducible nitric oxide synthase is actively involved in the synthesis of nitric oxide and contributes to the promotion of apoptosis (Vakkala et al., 2000). We proposed that an increase of NO production by the iNOS enzyme as the most likely mechanism by which nicotine and tobacco-specific nitrosamines of gutkha and khaini products could induce a chronic effect on the membrane and apoptosis.

## Materials and Methods

*Subjects*

Thirty human male volunteers in each group aged 27 ±5 residing in Ananthapuramu, India and taking local diet. The baseline information for the category of SLT users was that individuals used gutkha and khaini products (Khaleja/mahak chaini brand respectively) habitually, at least >20 times per week consists of 50-60 g during the last 2-4 years. Socio-demographic information was collected by an interviewer with the information on age, educational qualification, marital status, income, occupational status, and religion. Non-tobacco users, individuals do not consume any form of tobacco products. 

The inclusion criteria are the habitual use of only gutkha and khaini packets by the SLT users, and choose the unmarried and low economic status people. The exclusion criteria are the consumed either alcohol or smoking groups are not preferred. In the present study all volunteers were free from any chronic disease, illness, and teetotallers with no smoking habit with free from the use of any tranquilizers, drugs and anaesthetics. All experiments were performed in accordance with the approved guidelines and regulations of the Ethical Committee.

*Membrane studies*

Erythrocyte membrane was prepared by the (Dodge et al., 1963), platelets were prepared by differential centrifugation as described earlier by the method (Menashi et al., 1981), and membrane MDA levels by the method (Buege and Aust, 1978). The concentration of protein carbonyls was determined using 2, 4-dinitrophenylhydrazine (DNPH) assay (Levine et al., 1990), nitric oxide (Nitrites and nitrates) was estimated by (Sastry et al., 2002) and peroxynitrites were determined by (Beckman et al., 1992). Erythrocyte membrane lipids were extracted (Folch et al., 1951), estimation of cholesterol (Zlatkis et al. 1953), phospholipids (Connerty et al. 1961), protein concentration was measured by the method (Lowry et al., 1951), and quantitative measurement of membrane fluidity was performed by the fluorescence polarization technique described (Choi and Yu, 1990). The activity of glutathione was measured by the method (Ellman, 1959), superoxide dismutase (SOD) activity was measured according to the protocol (Kakker et al. 1984), estimation of catalase activity by the method (Aebis, 1974). Activity of GPx and GST were evaluated by the method (Rotruck et al., 1973).

*Erythrocyte membrane preparation*

Erythrocyte membranes were prepared using the method (Dodge et al., 1963). Erythrocyte suspension was washed with phosphate buffered saline (pH 7.2), and then cells were lysed with 5 mM phosphate buffer (pH 8.0) and spun at 15,000 X g for 30 min. The supernatant was removed carefully and by using the same buffer the latter step was repeated to obtain haemoglobin-free ghosts for further analysis.

*Osmotic fragility of erythrocytes*

Isolated red blood cells were incubated with different concentrations of NaCl ranging from 0.1% to 0.9% for 30 min with gentle stirring, hemoglobin released into supernatant from the red cells was determined after a spin at 2,500g for 10 min absorbance was measured at 540 nm by the method (Kanai, 1988).

**Table 1:** Levels of Biochemical Profile in Erythrocyte Membrane of Smokeless Tobacco Users

Parameter	Groups		
	Controls	Smokeless tobacco users	
		Gutkha chewers	Khaini chewers
Proteins (mg/dl)	131.78±7.02	105.78±7.35*	104.38±4.17*
Cholesterol (µg/mg protein)	160.35±11.72	257.34±24.68*	209.21±23.85^NS^
Phospholipids (µg/mg protein)	178.47±5.73	210.48±5.34*	191.34±4.89^NS^
C/P ratio	0.89	1.22	1.09

Data are represented as the mean ± SEM and

* denotes that data are significantly different with the normal controls. Note: NS- Not significant; C/P ratio- Cholesterol/ Phospholipids ratio.

**Table 2:** Biochemical Profile in Platelet Membrane

Parameter	Groups		
	Controls	Smokeless tobacco users	
		Gutkha chewers	Khaini chewers
Proteins (mg/dl)	83.98±4.36	91.55±4.06NS	87.74±4.63^NS^
Cholesterol (µg/mg protein)	373.77±26.75	588.74±23.71*	469.39±23.96**
Phospholipids (µg/mg protein)	127.40±5.30	163.44±5.04*	152.81±4.69*
C/P ratio	2.93	3.6	3.07

Data are represented as the mean ± SEM.

* denotes that data are significantly different with the normal controls and

** denotes that data are significantly different with the gutkha groups.

**Table 3:** Primers Used in Reverse Transcription Analysis

Gene	Primer Sequence
Bcl2	F5’CCTGATTCATTGGGAAGTTTCAA 3’
	R5’AAACAAATGCATAAGGCAACGA 3’
Bax	F 5’ TAATCCCAGCGCTTTGGAA 3’
	R 5’ TGCAGAGACCTGGATCTAGCAA 3’
TNF-α	F 5’GAAAGCATGATCCGGGACGTG 3’
	R 5’GATGGCAGAGAGGAGGTTGAC 3’
IL-6	F 5’-CCAGCTATGAACTCCTTCTC 3’
	R 5’-GCTTGTTCCTCACATCTCTC 3’
Caspase 8	F 5’-CTGGGAAGGATCGACGATTA 3’
	R 5’-CATGTCCTGCATTTTGATGG 3’
Caspase 10	F 5′-AATCTGACATGCCTGGAG-3’
	R 5′-ACTCGGCTTCCTTGTCTAC-3′
Caspase 12	F 5′-GCCATGGCTGATGAGAAACC-3’
	R 5′-CCTGAGTTGCTTCTTATGAG-3′
GAPDH	F 5’-GAGTCAACGGATTTGGTCGT-3’
	R5’-GACAAGCTTCCCGTTCTCAG-3’

**Figure 1:**
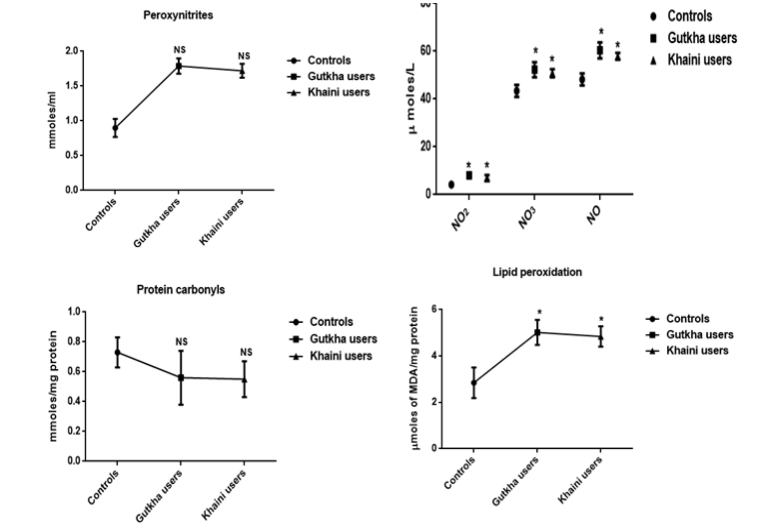
Levels of Oxidative and Nitrosative Stress Markers in Erythrocytes Data are Represented as the mean ± SEM. * denotes that data are significantly different with the normal controls. Note: NO2-Nitrites; NO3-Nitrates; NO-Nitric oxide; MDA-Malondialdehyde

**Figure 2:**
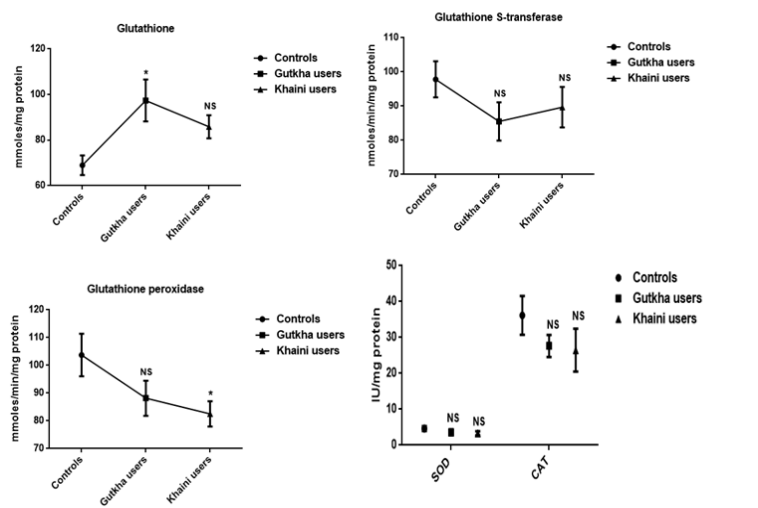
Status of Glutathione and Antioxidant Enzymes in Erythrocytes Data are Represented as the mean ± SEM. * denotes that data are significantly different with the normal controls. Note: SOD-Superoxide dismutase; CAT-Catalase

**Figure 3:**
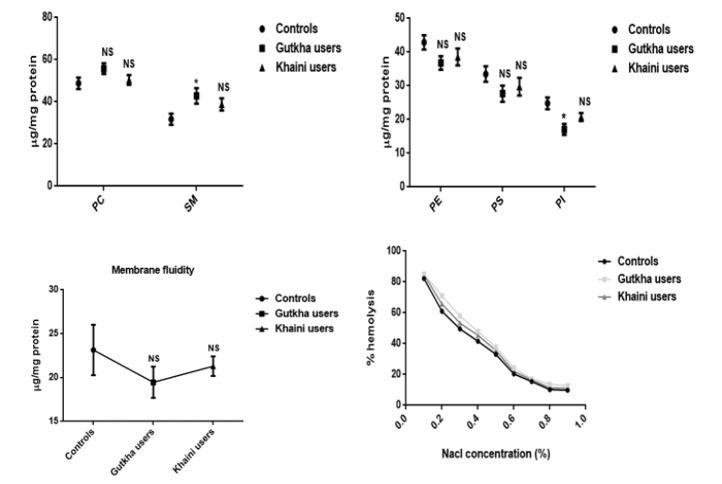
Concentrations of Individual Phospholipids and Membrane Fluidity in Controls and Smokeless Tobacco Users of Erythrocytes Data are Represented as the mean ± SEM.* denotes that data are significantly different with the normal controls. Results of osmotic fragility are expressed as mean. Note: PC-Phosphotidyl choline; SM-Sphingomyelin; PE-Phosphotidyl ethanolamine; PS-Phosphotidyl serine; PI-Phosphotidyl ionositol

**Figure 4:**
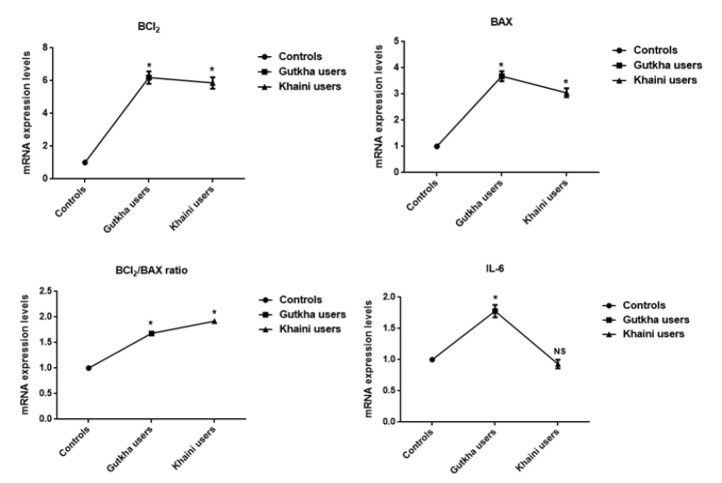
Effect of Smokeless Tobacco on Apoptotic Proteins Data are Represented as the mean ± SEM. * denotes that data are significantly different with the normal controls. Note: IL-6-Interleukin-6

**Figure 5:**
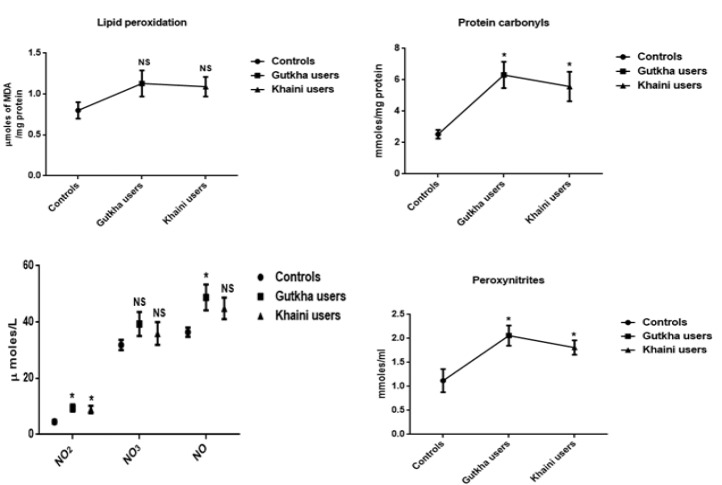
Concentrations of Oxidative and Nitrosative Stress Markers in Platelets Data are Represented as the mean ± SEM. * denotes that data are significantly different with the normal controls

**Figure 6:**
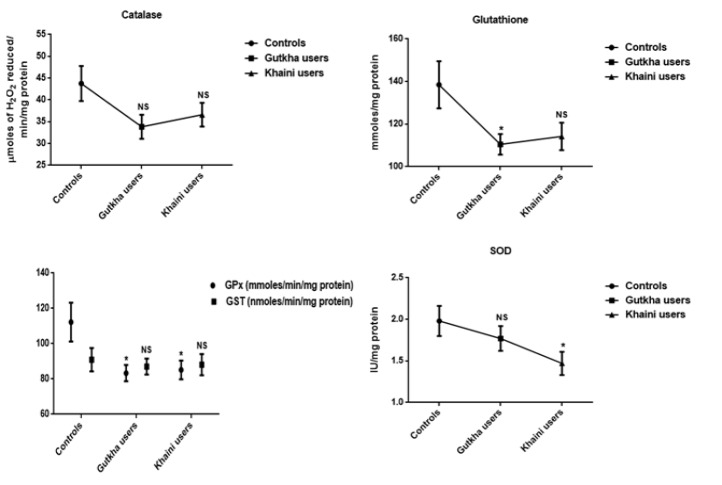
Antioxidant Enzymes in Platelet Membranes of Experimental Subjects Data are Represented as the mean ± SEM. * denotes that data are significantly different with the normal controls

**Figure 7:**
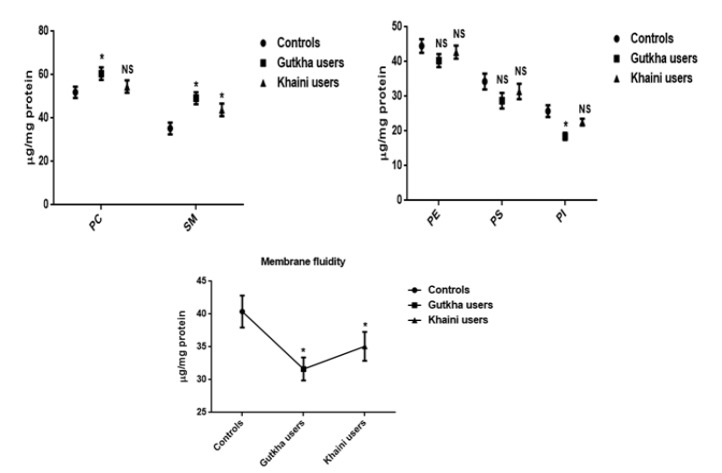
Smokeless Tobacco Induced Alterations in Individual Phospholipids and Membrane Fluidity in Platelets Data are Represented as the mean ± SEM. * denotes that data are significantly different with the normal controls

**Figure 8:**
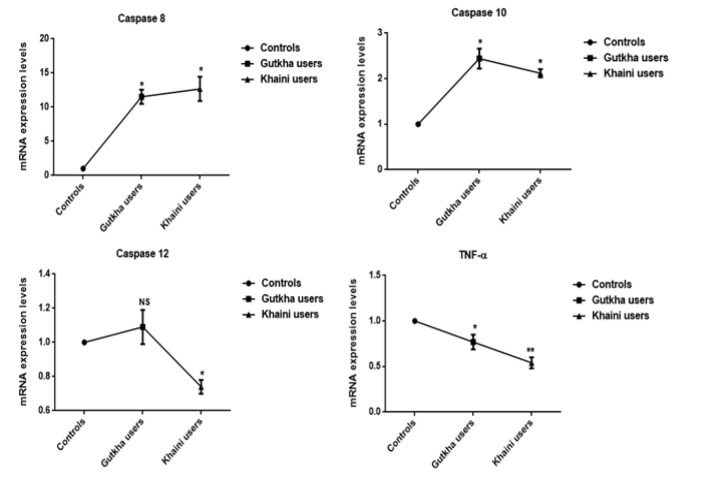
The Alterations of Different Caspases are Influenced by the Effects of Gutkha and Khaini Products Data are Represented as the mean ± SEM. * denotes that data are significantly different with the controls. Note: TNF-α-Tumor necrosis factor-alpha

**Figure 9:**
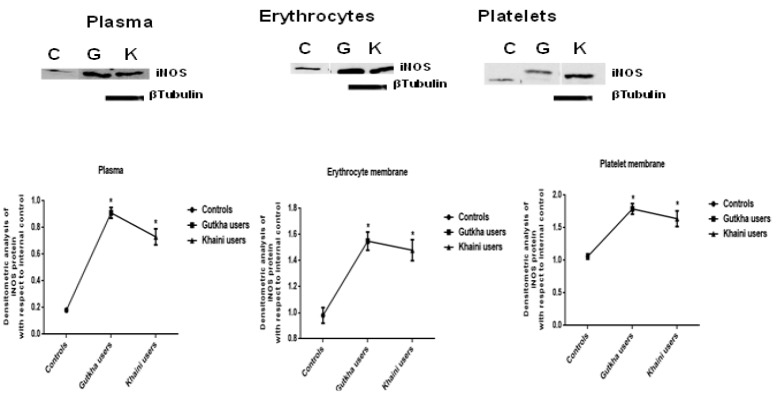
Immunoblots and Concentrations of Inducible nitric Oxide Synthase in Plasma, Erythrocytes, and Platelets Data are Represented as the mean ± SEM. * denotes that data are significantly different with the normal controls. Note: C-Controls; G-Gutkha users; K-Khaini users

**Figure 10:**
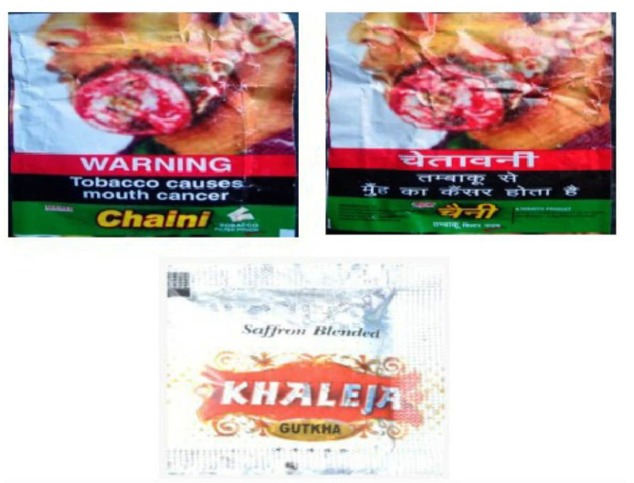
Photographs of Khaini and Gutkha Brands Available in Indian Tobacco Market

*Membrane individual phospholipids analysis*

Individual phospholipids in membranes of erythrocytes and platelets were estimated by the method (Skipski et al., 1964).The lipids were re dissolved in 1.0 ml of chloroform–methanol mixture and aliquots were used for the estimation of lipid components namely cholesterol and phospholipids. Erythrocyte membrane individual phospholipids were separated on silica gel H (Merck) using two dimensional thin layer chromatography with chloroform–methanol–aqueous ammonia 65:35:5 (v/v) as the first solvent and chloroform–acetone–methanol–acetic acid–water 50:20:10:10:5 (v/v) as the second solvent. The fractions were located with iodine vapours and scraped from the plate and the phospholipids were measured as inorganic phosphorus after digestion with perchloric acid method (Fiske and Subbarow, 1925).

*Western blot analysis*

Among the studied population thirty SLT-users and thirty non-users were randomly selected for molecular analysis. Immuno blots were performed from plasma, erythrocytes and platelet membranes to measure the level of iNOS and β -actin was used as positive loading control. Equal amount of protein (50 μg) was loaded in each lane followed by separation using sodium dodecyl sulfate-polyacrylamide gel electrophoresis (SDS-PAGE) and electro blotted on PVDF membrane (Millipore, Massachusetts, USA). The membrane was blocked for 4.0 h at 37°C with 5% bovine serum albumin (BSA) solution. Then the membrane was incubated with anti-rabbit polyclonal antibody (1:1000) for overnight at 4 °C, followed by incubation with alkaline conjugated anti rabbit antibody (1:2,500) for 4.0 h. After washing, the membrane was developed using ECL solution. All western blots were performed under the same experimental conditions.

*RNA extraction and cDNA synthesis*

Five micrograms of RNA per blood sample were separated on 1% formaldehyde-agarose gels to assess RNA integrity. RNA concentrations were amended for quality by spectophotometry (E=260 nm). First-strand cDNA was synthesized from 4 μg of total RNA using an Oligo(dT)12-18 primer and Superscript™ II RNase Reverse Transcriptase (Invitrogen, USA). Samples were stored at –20˚C.

*Measurement of mRNA from blood by RT-PCR analysis*

To determine apoptotic, interleukin, and TNF-α mRNA expression level of blood, total RNA was extracted using TRizol reagent from blood (Sigma) in accordance with the manufacturer’s protocol and kept at −70 °C for later use. The primer sequences of different genes are listed in Table 3.

## Results

*Biochemical analysis in membranes of red blood cells and platelets*

Data in table 1 and table 2, we presented that gutkha and khaini users have shown that significantly increased levels of cholesterol (P value; G =0.0018), phospholipids (G =0.054), and the C/P ratio in both erythrocytes and platelet membranes in comparison with normal healthy controls. The mean values of total membrane cholesterol (K =0.079), and total phospholipids (K =0.20) in khaini users did not showed significant change. The experimental users found to be decreased levels of membrane proteins in the red blood cells (G =0.051, K =0.012) and observed higher levels of platelet total proteins (G =, 0.21 K =0.56). 

*Concentrations of reactive oxygen nitrogen species (RONS) and antioxidants*

From the summary statistics in figure 1 it was observed that significantly increased levels of peroxynitrites (G =0.05, K =0.0001), nitrites (G =0.001, K =0.034), nitrates (G =0.038, K =0.023), nitric oxide (G =0.008, K =0.002), and lipid peroxidation (G =0.020, K =0.021) in erythrocyte membrane of experimental subjects compared to normal control subjects. We found that gutkha (G =0.45) and khaini users (K =0.30) have decreased levels of protein oxidation with no significant difference than controls. In this present study, smokeless tobacco users showed that decreased levels of superoxide dismutase (G =0.32, K =0.16), catalase (G =0.18, K =0.24), glutathione peroxidase (G =0.13, K =0.026), and glutathione S-transferase (G =0.12, K =0.31) in the erythrocytes. The levels of GPx exhibited statistically significant difference in khaini chewers. The experimental subjects have been shown that significantly increased levels of red blood cell glutathione (G =0.01, K =0.018) compared to normal controls (Figure 2).

*Individual phospholipids and fluidity studies*

In the current study, the mean difference of membrane fluidity for erythrocytes was decreased with no significant change observed in the experimental subjects (G =0.28, K =0.55). The increased levels of outer leaflets of phosphotidyl choline (PC) (G =0.07, K =0.065) and sphingomyelin (SM) (G =0.024, K =0.09) presented in the red blood cell membrane of smokeless tobacco users and the levels of sphingomyelin in gutkha users found to be significant change. The experimental subjects showed to be decreased levels of phosphotidyl serine (PS) (G =0.09, K =0.30), phosphotidyl ethanolamine (PE) (G =0.051, K =0.20), and phosphotidyl ionositol (PI) (G =0.004, K =0.06) in comparison with the normal subjects and mean values of phosphotidyl ionositol of gutkha groups showed significant difference (Figure 3).

*Gene expression analysis and status of oxidative stress*

We have obtained significant difference of increased concentrations of Bcl2, Bax, Bcl2/Bax ratio, and IL-6 were detected in the experimental subjects (Figure 4). The mean value of interleukin-6 was lower with no significant change in khaini users. The significantly increased levels of peroxynitrites (G =0.008, K =0.02), lipid peroxidation (G =0.10, K =0.08), protein oxidation (G =0.0003, K =0.002), nitrites (G =0.002, K =0.007), nitrates (G =0.12, K =0.36), and nitric oxide (G =0.01, K =0.054) were observed in the platelet membranes of habitual users of SLT. The mean difference of lipid peroxidation in SLT users, nitrates levels in gutkha users, and nitrates, nitric oxide in khaini users did not depicted significant difference (Figure 5). 

*Role of antioxidant enzymes in platelets*

Results obtained in this study, the decreased levels of glutathione (G =0.03, K =0.07), superoxide dismutase (G =0.42, K =0.046), glutathione peroxidase (G =0.02, K =0.03), glutathione S-transferase (G =0.62, K =0.75), and catalase enzymes (G =0.052, K =0.15) were present in the experimental subjects compared to controls. The mean values of glutathione peroxidase in SLT users, levels of GSH in gutkha users, and concentrations of SOD in khaini users exhibited statistically significant difference (Figure 6).

*Membrane fluidity and individual phospholipids*

The present study revealed that smokeless tobacco users reported that significantly increased levels of outer leaflets of PC (G =0.038, K =0.50) and SM (G =0.001, K =0.04) when compared to normal healthy controls and the mean values of PC in khaini users did not showed statistically significant change. The non-tobacco users found to be increased levels of PS (G =0.09, K =0.37), PI (G =0.001, K =0.12), and PE (G =0.13, K =0.51) were observed in the platelet membrane and the mean difference of PI has significant change in the gutkha users. The membrane fluidity showed significantly decreased levels was reported in habitual users of SLT (G =0.007, K =0.12) compared to normal control subjects (Figure 7). 

*Gene expression and western blot analysis*

Upon evaluation of the data (Figure 8) presented that the significantly increased concentrations of various caspases (Caspase 8, caspase 10, and caspase 12) and values of caspase 12 did not have any significant effect in gutkha chewers (G=0.36). The khaini chewers showed significant increase in the levels of caspase 8, caspase 10, and significant decreased expression of caspase 12. The smokeless tobacco users were found to be significantly decreased levels of TNF-α (G =0.012) than non-users group. The mean values of apoptotic proteins, TNF- α, and IL-6 showed significant change less than 0.05. Densitometric analysis of inducible nitric oxide synthase (iNOS) from the SLT-user group and the non-user group were represented in Figure 9. Moreover, we observed that smokeless tobacco users found to be significantly increased concentrations of iNOS in plasma, membranes of erythrocytes, and platelets (K=0.0002) in comparison with healthy controls. The levels of iNOS protein expression in SLT users of plasma, membranes of erythrocytes and platelets exhibited statistically significant difference. The brands of khaleja gutkha and mahak chaini of khaini were presented in Figure 10.

## Discussion

The consumption of smokeless tobacco is a public health threat and over 350 million users globally present in the South-East Asia region (Mehrotra et al., 2018). Free radicals are highly reactive species with unpaired electrons and could be able to involve in the damage of biomolecules like DNA strand breaks and enhanced mutation rates (Sun, 1990). The nitric oxide acts as pro-inflammatory agent when present in increased levels than normal physiological amounts (Sharma, 2007). Our results are reported that the significant increase in the levels of nitric oxide synthase through the production of nitric oxide in plasma, erythrocytes, and platelets. The enhanced production of superoxide anion, lipid peroxidation, DNA fragmentation in the primary cultures of human oral keratinocytes in response to smokeless tobacco exposure (Bagchi et al., 1999). The nitric oxide is involved in the form of dual role in induction or inhibition of apoptosis. In the presence of higher concentrations, it may involve in the enhancement of caspase proteins and release of cytochrome c (Choi et al., 2002).

From the summary statistics, we concluded that the significant increase in the concentrations of peroxynitrites (ONOO-) in the membranes of erythrocytes and platelets. The potential roles of protein oxidation and nitration, lipid peroxidation, mitochondrial dysfunction, and cell death, by the peroxynitrite represent both a pathophysiologically relevant endogenous cytotoxin and oxidant (Radi, 2018). ONOO- showed high rates of diffusibility and freely crosses through phospholipid membranes (Marla et al., 1997). Peroxynitrite is the oxidant which performed the oxidation of thiol groups present in cysteine and glutathione which required oxygen (Quijano et al., 1997). Peroxynitrite mediated nitration mechanisms and will serve to study the factors controlling protein and lipid nitration in bio membranes and lipoproteins (Bartesaghi et al., 2006).

The experimental subjects showed that the decreased levels of protein carbonyls in the erythrocytes, but in platelets enhanced rate of protein oxidation was observed in the smokeless tobacco users. The positive association of increased protein oxidation and cellular metabolism leads to oxidative stress (Cecarini et al., 2007). Excess cellular levels of ROS cause damage to bio molecules results in the enhancement of apoptosis (Redza-Dutordoir and Averill-Bates, 2016). The presence of smokeless tobacco exposure and chewing, habitual users exhibited that decreased activities of antioxidants in both erythrocytes and platelets compared to non-tobacco users. Previous reports revealed that decrease in H2O2 by down regulation of superoxide dismutase activity supports apoptosis (Kahl et al., 2004). The enzymatic antioxidants of superoxide dismutases, catalases, glutathione system, thioredoxin system, peroxidase systems, flavo hemoglobins and nitrate or nitrite reductases play an important role in the scavenging of excess ROS and RNS species (Staerck et al., 2017).

Data presented that the smokeless tobacco users exhibited that the significantly increased expression of Bcl2, Bax, caspase proteins, IL-6, and decreased expression of TNF-α in comparison with normal controls. Tumor necrosis factor-alpha (TNF-alpha) can cause DNA damages through reactive oxygen species and antioxidants significantly reduced TNF-alpha-induced genetic damage (Yan et al., 2006). Nicotine can induce the Bcl2 phosphorylation in association with prolonged survival through suppression of apoptosis (Mai et al., 2003). The smokeless tobacco extract (SLTE) caused dose-dependent cell death and reactive oxygen species production within 30 min to 3h of exposure (Mitchell et al., 2010). There is a positive uphill relationship between iNOS enzyme expression and apoptosis in human B cell lymphomas (Atik et al., 2006). The death ligands bind to death receptors results in recruitment of the caspase-8 which leads to the release of cytochrome c from mitochondria, triggering activation of apoptosis (Moodley et al., 2003).

From our results, we concluded that the significant rates of lipid peroxidation occurred in the membranes of gutkha and khaini users. The occurrence of free radical-induced lipid peroxidation causes considerable changes in the structural and functional organization of cell membrane (Jain, 1989). We have shown that the decreased membrane fluidity and increased osmotic fragility were detected in SLT users. The excess malondialdehyde is formed by the lipid peroxidation decreased the fluidity of the membrane lipid bilayer and increased the osmotic stability (Bryszewska et al., 1995). Increased HDL cholesterol levels may able to induce erythrocyte cholesterol:phospholipid ratios, resulting in decreased deformability and increased osmotic fragility (Meurs et al., 2005). Platelet total cholesterol was higher in users of smokeless tobacco and hyperactive platelets with elevated levels of cholesterol might be involved in the development of coronary artery disease (Ravindran and Krishnan, 2007).

Apoptosis is a highly organized form of programmed cell death play an essential role in tissue homeostasis, organ development and senescence. Reactive oxygen and nitrogen species can oxidize cellular glutathione leading to the loss of intracellular redox homeostasis and activation of the apoptotic signaling cascade (Circu and Aw, 2012). The mean values of erythrocyte membrane cholesterol have found to be increased levels in gutkha and khaini users. Abnormal erythrocyte morphology was observed in hypercholesterolemic mice and morphology appeared immature with irregular shape (Holm et al., 2002). Increased membrane cholesterol could be involved in the inhibition of apoptosis (Verstraeten et al., 2018). Fluidity of the membrane is decreased in diabetic patients and may complicate the flow of these cells in micro vessels (Shin et al., 2007).

In summary, we concluded that the role of reactive oxygen and nitrogen species, decreased activities of antioxidant enzymes like SOD, up regulation of nitric oxide synthase, increased expression of caspase 8 proteins compared to antiapoptotic protein Bcl2 played a major role in the activation and progression of apoptosis in the blood of exposed gutkha and khaini users. The smokeless tobacco products are actively engaged in the elevation of apoptosis in the blood and further studies are needed to investigate the specific toxicants of SLT products in the induction of apoptosis.
